# Supplementary material for: The interactome of intact mitochondria by cross-linking mass spectrometry provides evidence for coexisting respiratory supercomplexes
Source: Mol Cell Proteomics. 2017 Dec 8;17(2):216–32. doi: 10.1074/mcp.RA117.000470 (PMC5795388; doi:10.1074/mcp.RA117.000470)
Supplement: Supplemental Data [file supp_RA117.000470_133922_0_supp_23978_fzffwf.zip › spectra_annotation/mito_spectra_annotation/53-1-6-1-7-1.pdf]

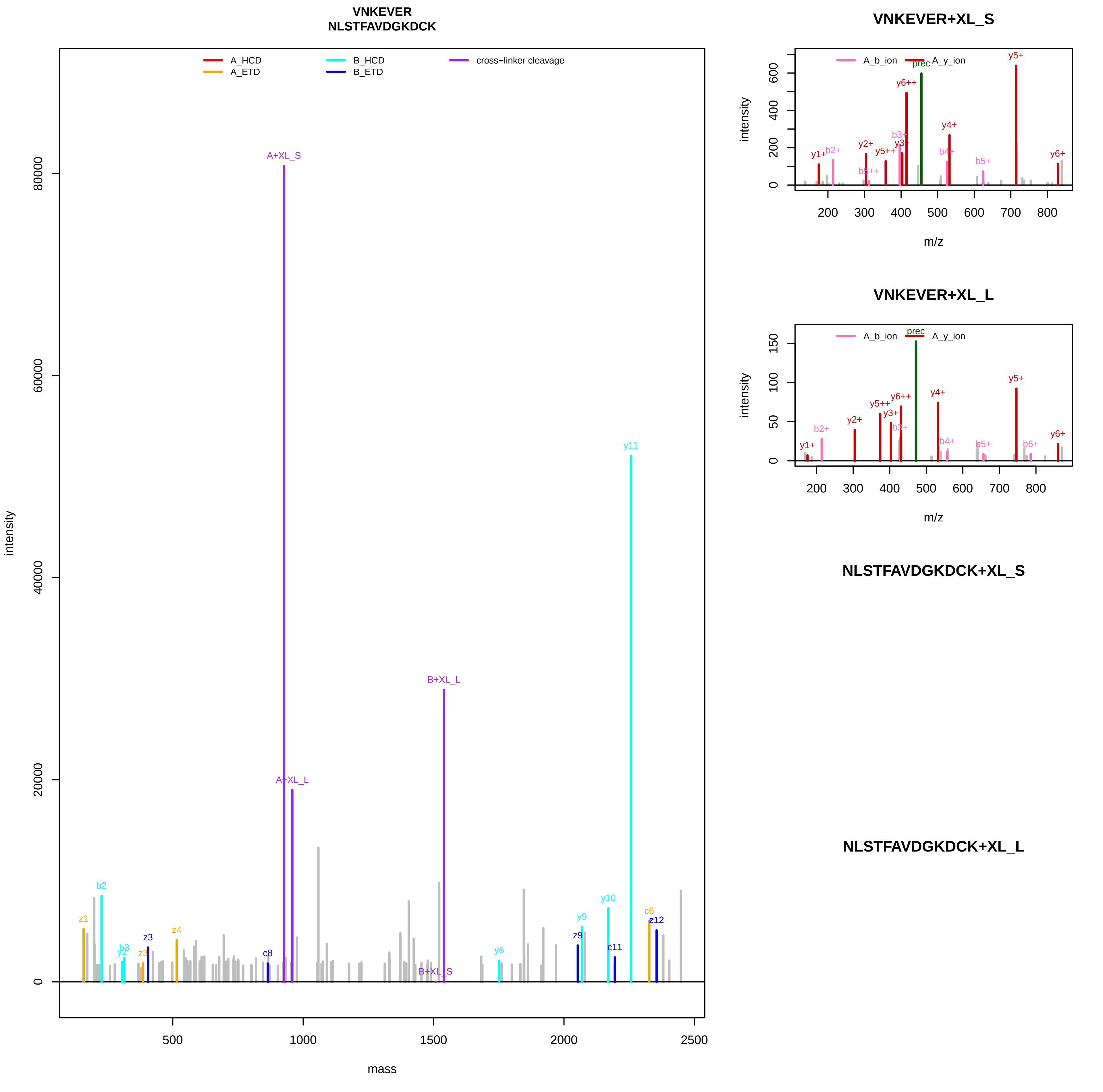
VNKEVER
NLSTFAVDGKDCK
A_HCD
A_ETD
B_HCD
B_ETD
cross−linker cleavage
A+XL_S
A+XL_L
B+XL_L
B+XL_S
y11
y10
y9
y6
y2
b2
b3
z1
z3
z4
z9
c6
c8
c11
c12
intensity
mass
0
20000
40000
60000
80000
500
1000
1500
2000
2500
VNKEVER+XL_S
A_b_ion
A_y_ion
prec
y1+
b2+
y2+
b3++
y5++
b3+
y3+
y6++
y4+
b4+
b5+
y5+
y6+
intensity
m/z
0
200
400
600
800
700
300
100
VNKEVER+XL_L
A_b_ion
A_y_ion
prec
y1+
b2+
y2+
y5++
y3+
y6++
b3+
y4+
b4+
b5+
y5+
b6+
y6+
intensity
m/z
0
50
100
150
NLSTFAVDGKDCK+XL_S
NLSTFAVDGKDCK+XL_L
